# Sorafenib Resistance in Hepatocellular Carcinoma: The Relevance of Genetic Heterogeneity

**DOI:** 10.3390/cancers12061576

**Published:** 2020-06-15

**Authors:** Loraine Kay D. Cabral, Claudio Tiribelli, Caecilia H. C. Sukowati

**Affiliations:** Fondazione Italiana Fegato (Italian Liver Foundation), AREA Science Park, Basovizza, 34149 Trieste, Italy; kay.cabral@fegato.it (L.K.D.C.); ctliver@fegato.it (C.T.)

**Keywords:** hepatocellular carcinoma, drug resistance, sorafenib, tumor heterogeneity

## Abstract

Despite advances in biomedicine, the incidence and the mortality of hepatocellular carcinoma (HCC) remain high. The majority of HCC cases are diagnosed in later stages leading to the less than optimal outcome of the treatments. Molecular targeted therapy with sorafenib, a dual-target inhibitor targeting the serine-threonine kinase Raf and the tyrosine kinases VEGFR/PDGFR, is at present the main treatment for advanced-stage HCC, either in a single or combinatory regimen. However, it was observed in a large number of patients that its effectiveness is hampered by drug resistance. HCC is highly heterogeneous, within the tumor and among individuals, and this influences disease progression, classification, prognosis, and naturally cellular susceptibility to drug resistance. This review aims to provide an insight on how HCC heterogeneity influences the different primary mechanisms of chemoresistance against sorafenib including reduced drug intake, enhanced drug efflux, intracellular drug metabolism, alteration of molecular targets, activation/inactivation of signaling pathways, changes in the DNA repair machinery, and negative balance between apoptosis and survival of the cancer cells. The diverse variants, mutations, and polymorphisms in molecules and their association with drug response can be a helpful tool in treatment decision making. Accordingly, the existence of heterogeneous biomarkers in the tumor must be considered to strengthen multi-target strategies in patient-tailored treatment.

## 1. Introduction

### 1.1. Hepatocellular Carcinoma: Obstacles in Conventional Treatment

Hepatocellular carcinoma (HCC) is a heterogeneous malignancy primarily affecting the hepatocytes. It had an annual incidence of about 841,000 new cases worldwide in 2018 and ranks as the sixth most common malignancy and fourth most common cancer-related death in the world [1,2]. The incidence of HCC is associated with its known diverse underlying etiologies that reflect geographical distribution. In Eastern Asia and Africa, the highest factor is a chronic infection of hepatitis B virus (HBV), whereas in Western countries and Japan, chronic infection of hepatitis C virus (HCV) is the highest risk factor [3], together with excess alcohol intake and metabolic syndrome. Despite numerous studies for an early diagnosis, the treatment for HCC remains one of the most difficult to cure [4] and is described as a “chemoresistant” tumor [5]. The carcinogenesis complexity increases the burden in the diagnosis while the heterogeneity (tumor extent, patient comorbidities, and severity of liver dysfunction) challenges both management and treatment [6]. While proven to be potentially curative and improving survival, radical treatments such as surgical resection and liver transplant are considered only for early-stage HCC [7], which accounts for a small number of HCC cases. Complete surgical removal is not an option for the majority of HCC patients since more than two-thirds of its cases are already in the advanced and metastatic stages at the time of diagnosis [8]. Besides, more than 90% of HCC patients have an incidence of post-surgery recurrence [9]. Radiofrequency ablation (RFA) and transarterial chemoembolization (TACE) are options for unresectable HCC cases [10,11]. Both are locoregional techniques that induce necrosis resulting in tumor shrinkage. For TACE treatment, the coupling with targeted delivery of cytotoxic chemotherapy (e.g., doxorubicin, cisplatin, epirubicin) increases tumor response, decreases progression, and improves overall survival [12,13]. However, these available treatments have remained very limited and only a handful can benefit from existing anti-neoplastic therapies. With only 15% of HCC eligible for the potentially curative treatments [14], the majority of HCC patients are in the advanced stage and relies on modest benefits of targeted treatments. Despite the decade of progress in improving treatment modalities for HCC [15], there still remains a challenge in overcoming toxicity and chemoresistance.

### 1.2. Molecular Therapy with Sorafenib

Sorafenib remains the globally accepted systemic first-line treatment for advanced HCC [16]. Even though it only has modest improvement in over-all median survival, its approval in 2007 is one of the hallmarks of HCC treatment. Sorafenib is a molecularly-targeted agent that works on the vascular endothelial growth factor receptors (VEGFR1, 2, 3), platelet-derived growth factor receptor-β (PDGFRβ) and the Raf family kinases (predominantly C-Raf rather than B-Raf) [17]. Two international randomized controlled trials (RCT) were pivotal in the approval of sorafenib treatment for advanced HCC. First was the Sorafenib HCC Assessment Randomized Protocol (SHARP), where 602 patients were randomized to receive sorafenib or placebo therapy. The sorafenib treated group showed an improved median overall survival of approximately 3 months compared to the placebo group (10.7 vs. 7.9) [16]. While the SHARP trial was limited to Caucasians, most related to HCV infection, a different study was conducted involving Asia-Pacific patients with underlying HBV infection, advanced HCC, and worse liver function. On the contrary, the result of the Asia-Pacific trial showed a marginal improvement in median overall survival (6.5 vs. 4.2 months, sorafenib vs. placebo) [18]. Although with limited efficacy, both RTCs indicated sorafenib as the standard therapy treatment agent for first-line therapy of patients with advanced HCC. They also showed different sensitivity to the treatment in approximately 30% of patients [16,18] indicating inherent or acquired resistance to the drug [19]. 

The standard approved dosing for the treatment of sorafenib is 400 mg twice daily (800 mg/day) [16]. Some physicians consider treating patients with a lower starting dose to reduce treatment costs and to manage toxicity without compromising efficacy [20]. The retrospective study of Reiss et al [21] showed that patients treated with a reduced dose vs those treated with a standard dosage of sorafenib did not result in inferior over-all survival but reduced treatment costs, and decreased the rate of adverse events. While dosing strategies can contribute to improving anti-tumor efficacies of sorafenib, the management of its resistance is still complex and needs further understanding and validation.

## 2. Mechanisms of Sorafenib Resistance and HCC Heterogeneity

It is known that human DNA polymorphisms and mutations are associated with risk and susceptibility to cancer development, including HCC. Besides a classical morphological classification, HCC heterogeneity was classified by highly comprehensive ‘-omics’ analysis. Based on global transcriptome analyses, gene mutations, promoter methylation, and HBV DNA copy number, Boyault et al. classified HCC based in six subgroups (termed G1 to G6) [22]. Specific aberrant molecular pathway profiles in the Wnt/β-catenin pathway were observed in each subgroup while a high-throughput sequencing and gene expression profiling identified distinct transcriptomic subclasses and numerous recurrent genetic alterations [23], including for various genes and proteins involved in drug resistance. Drug resistance is caused either by primary resistance (before drug exposure) or acquired resistance (after drug exposure). Both consist of a complex mechanism of chemoresistances (MOCs), including reduced drug intake, enhanced drug efflux, intracellular drug metabolism, alteration of molecular targets, activation/inactivation of signaling pathways, changes in DNA repair machinery, and negative balance between apoptosis and survival of the cancer cells [24].

Previous studies analyzing different somatic mutational profiles in HCC described mutations in the TERT promoter, TP53, CTNNB1 (encodes β-catenin), ARID1A, and AXIN1. Deep-sequencing studies also confirmed frequent TP53 and CTNNB1 mutations in HCC and pointed to novel HCC-associated mutations in genes involved in chromatin remodeling, ubiquitination, RAS/MAPK signaling, oxidative stress, and the JAK/STAT pathway [25,26,27,28]. The presence of somatic mutations in HCC can be further stratified by the prevalence of the geographical-associated risk factors. A study on the comparison of HCC somatic mutations between Asian and European ancestry in the TCGA Data Portal showed that TP53 and RB1 were mutated at significantly higher frequency in Asian than in European ancestry. This high frequency was also noticed for the VEGF-binding pathway (12% vs 2%) [29], a molecular target of sorafenib. 

Subsequently, the role of such mutations and variants to HCC drug response were also investigated. In an exploratory study, next-generation sequencing (NGS) of 17 regorafenib-treated patients revealed variants in oncogenes and tumor suppressor genes, mostly in the promoter region of TERT and TP53. Interestingly, CTNNB1 mutations were only observed in non-responder patients, while VEGFA amplification was only observed in responders. Despite its small sample size, the findings warrant further investigation since these markers are known to be key players of HCC carcinogenesis and drug response [30]. Using 126 proteins in 34 liver cancer cell lines, a recent study demonstrated genetic alterations and gene expression patterns associated with the response to 34 anticancer agents (including trametinib, sorafenib, and FGFR4 inhibitors). These molecular characterizations might be used to select patients for clinical trials [31] to possibly improve the therapeutic response. 

### 2.1. Drug Efflux: ABC Transporters Family

Cancer cells have various defensive mechanisms upon exposure to anti-neoplastic drugs. The proteins responsible for the removal of drugs from the cytoplasm maintaining non-toxic intracellular concentration is one of the most significant contributors of such defense. The ATP-binding cassette (ABC) transporters are one of the largest families of membrane transport proteins. These proteins utilize a pair of ATP (Adenosine-5’-triphosphate) molecules to export specific compounds or to flip them from inner to outer leafs of the membranes. In humans, there are 49 members of ABC transporters gene which are classified into seven subfamilies based on the sequence homology and ATP-binding proteins [32]. Genetic variants are widely noticed in DNA sequences of ABC transporters genes. A recent human genetic high throughput study by next-generation sequencing (NGS) in 138,000 individuals across seven major populations showed the extension of the population-specificity of ABC variants [33]. For drug-transporter members of the family, this study found clinically related variants for ABCB family, ABCC family, and ABCG2, which was already associated with chemotherapy resistance. The functional significance of single nucleotide polymorphisms (SNPs) of ABCB1 (PGP/MDR1), ABCC1 (MRP1), ABCC2 (MRP2), and ABCG2 (BCRP) in in vitro systems, in vivo, and drug disposition was previously reviewed, as well as on the clinical outcome [34,35]. Various SNPs of the ABCB1 (SNPs 335T>C, 3073A>C, 3751G>A, and 4125A>C) had also been associated with the risk of occurrence of HCC in Chinese populations [36,37,38]. 

Initially, the activation of ABC transporter proteins was observed upon exposure to conventional chemotherapy agents such as doxorubicin, cisplatin, and 5-fluorouracil (5-FU) as their common substrates [39,40,41]. In the development of molecular therapy, sorafenib was shown to modulate multidrug resistance caused by chemotherapy. The addition of sorafenib to conventional chemotherapy restored the chemosensitivity in highly resistant HCC. Doxorubicin plus sorafenib was demonstrated to reduce the mRNA levels of PGP, MRP1, MRP2, and MRP3 compared to doxorubicin monotherapy [42]. In contrast, ABCG2 (BCRP) was found to mediate the efflux of sorafenib; where co-treatment of sorafenib with an ABCG2 inhibitor greatly augmented the cytotoxicity in HCC cells [43]. This sorafenib resistance might be attributed to the genetic polymorphism in ABCG2. SNPs of ABCG2 1143 C>T and ABCG2 34 G>A were found to be significantly associated with the lowest sorafenib plasma levels and better clinical outcome [44,45]. ABCB1 3435 C>T genotype was also related to sorafenib sensitivity in HCC patients [44]. 

A recent study showed a possible association between sorafenib resistance and ABCC2 (MRP2) variants. By analyzing 110 individuals, a substantial inter-individual variability of transporter expression in a normal liver, including high MRP2 expression, was correlated with the 3600A and 4581A ABCC2 variants [46]. ABCC2 polymorphisms were found to be related to ATPase activity. In an in vitro study, cells overexpressing the ABCC2 1249A allele showed lower intracellular accumulation of sorafenib than in cells ABCC2 1249G. Isolated ABCC2 1249A protein showed higher ATPase activity than ABCC2 1249G protein [47]. This result was in line with a previous study using different ABCC2 SNPs generated using site-directed mutagenesis targeting C24T-, G1249A-, G3542T-, T3563A-, C3972T- and G4544A-MRP2. Genetic variability in the ABCC2 gene was found to influence the in vitro expression, trafficking, and transport activity of MRP2 [48]. High efflux of chemotherapy drugs as paclitaxel and doxorubicin was also noticed in ABCC2 1249A polymorphism [49]. 

### 2.2. Drug Uptake: The SLC Family

The human solute carrier (SLC) superfamily of transporters consists of 400 membrane-bound proteins with important roles in physiological processes, ranging from the cellular uptake of nutrients to the absorption of drugs and other xenobiotics, including cancer drugs. These transporters utilize an electrochemical potential difference or an ion gradient for transporting their substrates across biological membranes [50,51]. Hence, their role in determining cellular response to cancer therapy is essential. However, compared with the abundant data available on ABC-transporter proteins, the function of SLC transporters in molecular therapy resistance has only been recently investigated. The SLC superfamily is genetically heterogeneous. A recent study by Schaller et al. using a bioinformatics analysis on NGS data from around 140,000 individuals from seven major human populations resulting in more than 200 exonic single-nucleotide variants. Most of the SLC variants were ethnic-specific and they were associated with clinical drug response or toxicity phenotypes [51]. The polymorphic properties of SLC members such as OCT1 is associated with genetic heterogeneity in global populations and has therapeutic relevance [52].

Several studies indicated the importance of the member 22 of the solute carrier family (organic cation/anion transporter) in sorafenib resistance. SLC22A1 (OCT1) has been reported to be involved in the uptake of sorafenib, both in an animal model or human cells [53,54], even though a recent study showed opposing results [55]. Downregulation of OCT1 and OCT3 (SLC22A3) was observed in HCC [56,57] which was further associated with a poor survival in patients treated with sorafenib, independently from the clinical staging of the associated liver disease [58]. Schaeffeler et al. reported that DNA methylation of OCT1 is associated with OCT1 downregulation [59], suggesting that targeting OCT1 methylation by demethylating agents would be useful for the management of sorafenib-resistant patients.

Several OCT1 variants (R61C, C88R, S189L, M420del, and G465R) were shown to reduce the translocation efficiency of lamivudine, an antiviral drug, and affected drug–drug interaction [60]. In contrast, variants such as S29L, C88R, E284K, G465R, R61C, and R206C show reduction/loss of the transport activity of OCT1 to its substrates and, hence, could be valuable in improving drug uptake activity [52,61]. In addition, novel variants R61S fs*10 and C88A fs*16 showed abolished transport ability of OCT1 to sorafenib due the production of truncated protein. Even though these two variants were only found in less than 20% of HCC specimen, its presence may contribute to the intracellular concentration of sorafenib contributing to reduction in treatment efficacy [54]. However, in terms of increased sorafenib uptake, OCT1 variants such as S14F, L160F, G401S and P197S demonstrate stimulated activity [54].

Besides OCT1, a study using human hepatocytes and OCT1-transfected cell lines suggested that other organic anion transporting polypeptide(s) might be also involved in sorafenib uptake [62]. The expression of SLC46A3 (FKSG16) was downregulated in around 80% of human HCC tissues compared to non-tumor adjacent tissues, while tumors that expressed low levels of SLC46A3 had more aggressive phenotypes and shorter survival times after surgery [63]. HCC cells that stably overexpressed SLC46A3 inhibited the levels of migration and invasion compared with control HCC cells, and formed smaller xenograft tumors with more metastases. SLC46A3 overexpression could reduce sorafenib resistance and improve drug response both in vitro and in vivo models [63]. 

### 2.3. Alteration of Molecular Targets 

Sorafenib has the dual-target inhibition function to block the serine-threonine kinase Raf, which is part of the Ras/MEK/ERK signaling pathway, and to inhibit the tyrosine kinases VEGFR/PDGFR. Thus, DNA variants or mutations in these sites would affect the efficiency of the treatment. Elevated tissue expression of pERK and VEGFR-2 was predictive of poor outcome in advanced HCC treated with sorafenib [64,65]. SNPs in VEGFR2 gene (in particular rs2034965) were significantly associated with clinical outcomes of HCC patients [66]. 

HCC expresses a high level of VEGFA, a crucial regulator of tumor vascularization. Various isoforms of VEGFA (in particular VEGFA-165) have been related to invasiveness, disease status, and HCC development [67,68,69]. In a mouse model of inflammation-driven cancer and in HCC patients, HCC subclass carrying VEGFA amplification was particularly responsive to VEGFA inhibition and sorafenib treatment [70]. Furthermore, in ALICE-1 study, SNPs analysis of VEGFA, VEGFC, and VEGFR-1,2,3 from 148 samples receiving sorafenib showed that VEGFA allele C of rs2010963 and VEGFC allele T of rs4604006 together with Barcellona Clinic Liver Cancer (BCLC) stage were independent factors influencing progression-free survival (PFS) and overall survival (OS) of HCC patients. Several other SNPs in three targets were also significant predictors of PFS and OS [71]. 

A comprehensive analysis of biomarkers (BIOSTORM) was performed in 83 HCC patients receiving sorafenib compared to 105 receiving placebo in the 2019 phase 3 STORM study. The study included gene expression profiling, targeted exome sequencing, immunohistochemistry on pERK, pVEGFR2, Ki67, fluorescence in situ hybridization of VEGFA and immunome profiling. A 146-gene signature (87 poor + 59 good prognosis genes) was described to discriminate patients benefiting from sorafenib. It allowed the identification of 30% of patients who might benefit from sorafenib. Multivariate analysis showed that a poor PFS was correlated only with hepatocytic pERK expression and the presence microvascular invasion [72]. In line with the 3 STORM study, Harding et al. analyzed 341 cancer-associated genes using a hybridization capture-based NGS assay from 127 patients (81 received sorafenib). They found that patients whose HCCs contained activating PI3K/mTOR mutations exhibited a poor clinical outcome compared to patients without the mutations. However, mutations predicted to activate the WNT or MAPK pathway (including alterations in tyrosine kinases receptor), TP53 pathway, cell-cycle control, and chromatin remodeling did not affect the clinical outcome. VEGFA amplification, a proposed biomarker for extreme sorafenib responders was rarely observed and did not appear associated with clinical benefit [73]. In contrast, in 31 HCCs patients treated with immune checkpoint inhibitors, the activation of WNT/β-catenin signaling was associated with a poor response and shorter survival [73].

As a multi-kinase inhibitor, sorafenib also targets other kinases like the BRAF (B-Raf proto-oncogene). It is a family of serine/threonine protein kinases that plays a role in regulating the MAP kinase/ERK signaling pathway. Several reports showed that BRAF mutations were correlated with a response to the drug. In non-small-cell lung cancer (NSCLC), BRAF G469R mutation showed a dramatic response to sorafenib [74], while other variant G469V could also affect sorafenib sensitivity in HCC [75]. 

### 2.4. Alteration of Signaling Pathways

One of the most important factors in hepatocarcinogenesis is the modulation of developmental and oncogenic signaling pathways involved in the pathogenesis of HCC, such as the key signal transduction pathways Wnt/β-catenin, EGFR-RAS-MAPKK, c-MET, IGF signaling, Akt/mTOR signaling, and VEGF and PDGFR signaling cascades [76]. Sorafenib has directed its activity to important pathways such as the Ras/Raf/MEK/ERK signaling pathway and inhibits angiogenesis through the targeting of the hepatocyte factor receptor (c-Kit), Fms-like tyrosine kinase (FLT-3), VEGFR1,2,3, PDGFR-β, and other tyrosine kinases [17,77]. Therefore, genetic mutations in the major cellular signaling pathways may underlie the development of resistance to molecular targeting agents.

The epidermal growth factor receptor (EGFR/ErbB1/HER-1) was observed mainly in the cell membrane and occasionally and faintly in the cytoplasm of HCC. EGFR was expressed in the majority of HCC clinical samples and it was correlated with proliferating activity, stage, intrahepatic metastasis, and carcinoma [78]. The most common EGFR variant, EGFRvIII (de2-7 EGFR) promoted tumor cell growth and inhibited cell sensitivity to 5-FU [79]. Signaling pathways controlled by EGFR and HER-3 could restrict sorafenib effects both in naive and sorafenib-resistant HCC cells [80]. Since more than half of HCC patients have EGFR overexpression and abnormal activation [77], the combination of the EGFR inhibitor with HCC conventional chemotherapy agents are potential strategies to improve efficacy to treatment [81].

Ezzoukhry et al. highlighted the important differences in the sensitivity of different HCC cells to sorafenib. Some cell lines are more resistant to sorafenib compared to others. In sorafenib-resistant hepatoma cells, the efficacy of the treatment was increased when EGFR was inhibited by drugs (erlotinib or gefitinib), a monoclonal antibody directed against EGFR (cetuximab), and RNA interference [82]. These data suggest that the overexpression of EGFR may lead to sustained activation of EGFR downstream signaling and drug resistance to sorafenib [77,82]. EGFR downstream-signaling molecules, especially Ras/Raf/MEK/ERK signal transduction pathways, were also related to sorafenib resistance. The extent of sorafenib inhibition to the ERK phosphorylation (pERK) was significantly based on cell basal pERK expression level [83]. Variants of EGFR played an important role in p21 regulation and were associated with the clinical outcome of HBV-related HCC in a TP53-independent manner [84]. EGFR variants are reported to be correlated with drug sensitivity. HCC-derived EGFR mutants (K757E, N808S, R831C, V897A, P937L, T940A, and M947T), erlotinib-sensitive (L858R) mutants, and erlotinib-resistant mutants (T790M) were constructed by transfection. Erlotinib induced a differential degree of apoptosis and autophagy among cells harboring different EGFRs [85]. 

### 2.5. Intracellular Drug Metabolism

Sorafenib has a mean half-life ranging between 25 to 48 h. In healthy volunteers, following oral administration, approximately 19% of the dose is excreted in the urine, almost exclusively as glucuronide conjugates, and 77% in feces (50% unchanged). In the cell, sorafenib is metabolized by two pathways: phase I oxidation mediated by P450 enzymes (CYPs), and phase II conjugation mediated by UGT (uridine diphosphate glucuronosyltransferase) 1A9 (UGT1A9) to form the M1-M8 metabolites [86]. Information on the association between sorafenib and metabolic enzyme polymorphisms is very limited and mainly focus on its toxicity. A 2018 study by Guo et al. showed a minimal sorafenib metabolism in individuals with CYP3A5*3 genotype, which was further confirmed in an animal model [87]. It should be noted that broad genetic variations in P450s vary among different ethnic groups and its expression may affect drug metabolism activity [88]. Polymorphism in UGT1A9 (rs17868320) identified patients at high risk for early sorafenib-induced severe toxicity [45], while UGT1A1 (rs8175347) was associated with sorafenib side effects [89]. A 2012 study showed that the genotype status of UGT1A9 (also UGT1A1 and ABCC2) and serum bilirubin concentration was indicated with sorafenib metabolism. Patients carrying UGT1A1*28/*28 and UGT1A9*3/*3 have a higher area under the curve (AUC) than those patients corresponding to any other genotypes [90]. 

### 2.6. DNA Repair Pathway

Carcinogenesis is commonly associated with an elevated level of DNA-repair capacity leading to chemo- and radio-resistance. A sustained capacity of a cancer cell to perform DNA repair despite disruptive actions of chemotherapeutic drugs will lead to cell survival and growth, promoting resistance [91]. One DNA repair protein, the apurinic/apyrimidinic endonuclease/redox effector factor 1 (APE1/Ref-1) is a multifunctional protein playing a central role in the DNA base excision repair (BER) pathway of DNA lesions [92]. In HCC, APE1/Ref-1 protein was over-expressed in both the tissue and serum. Cytoplasmic localization was associated with a low degree of cancer differentiation and short survival time, indicating a prognostic function [93,94]. Besides its DNA repair function, the molecular basis of transcriptional regulation of APE1 is associated with drug resistance. APE1, preferably in the acetylated form, stably interacts with Y-box-binding protein 1 (YB-1) and enhances its binding to the Y-box element, leading to the activation of the MDR1 [95]. Loss of APE1’s acetylation impairs MDR1 activation and sensitizes the cells to cisplatin or etoposide [96].

APE1/Ref-1 polymorphism is present in human populations, and non-synonymous APE1 variants may act as cancer susceptibility alleles. A stratified analysis showed that individuals with the APE1-148-combined genotype GT+TT likely had a significantly higher HCC risk compared with those with only the GG genotype [97]. It was demonstrated that APE1 D148E, D283G, L104R, and R237C variants have different endonuclease activity and ability to associate with XRCC1 and DNA polymerase β, which are enzymes acting downstream of APE1 in the BER pathway. It may lead to a dysregulation of the DNA damage response [98]. A recent study using multiple datasets from the Oncomine, GEO, and TCGA databases showed that high APE1 gene expression correlated with the resistance to sorafenib in HCC patients. It was also associated with poorer overall and disease-specific survival, progression-free survival, and relapse-free survival in early- and advanced-stage HCC patients [99]. Another study showed that conformational dynamics and catalytic activities of APE1 variants affected the enzymatic process and catalytic activity [100]. APE1 D148E is one of the most studied variants. APE1 D148E, together with XRCC1 R194W polymorphism plays a role in the cisplatin resistance of HCC cells [101]. Conversely, other studies showed disagreement between the association between APE1 D148E and the risk of HCC development [97,102]. 

### 2.7. Autophagy 

Autophagy, a self-digestion capacity for the elimination of cytoplasmic materials, cellular components, and organelles, is one of the key factors for cell homeostasis in normal physiology. Depending on the type of cell and initiating stimulus, the role of autophagy can either be for cell survival or cell death [103]. In HCC, sorafenib induces autophagy and autophagic flux as a survival mechanism to resist sorafenib anti-cancer effects. Both in vitro and in the xenograft model sorafenib led to the accumulation of autophagosomes, as evidenced by conversion from LC3-I to LC3-II protein [103]. A study in HepG2 cells demonstrated that the upregulation of p62 (SQSTM1) was correlated with the reduction in sorafenib sensitivity. These data were in agreement with the observation that human HCC cases showing the expression of P62 were associated with drug resistance and shorter survival time [104]. However, the exact mechanisms underlying autophagy and sorafenib resistance remain unclear [105]. 

Recent studies indicated that genetic variants in autophagy-related genes (ATGs) could also affect the development of HCC. ATG5, together with ATG16L1 and ATG12, plays a critical role in autophagosome formation and elongation [106]. A recent study showed that sorafenib-induced autophagy was reduced by the upregulation of miR-142-3p that targeted ATG5 and ATG16L1 [107]. The formation of Atg5-deficient autophagosomes in response to sorafenib promoted the interaction of p62 with RIPK leading to cell death by necroptosis [108]. Several ATG5 variants were associated with the disease progression and HCC risk in chronic HBV infection [109,110]. A Chinese comparative study comprising around 1000 HCC cases vs. 1000 healthy controls showed that in an allelic model, ATG variants ATG5 rs17067724, ATG10 rs1864183, ATG10 rs10514231, ATG12 rs26537, and ATG16L1 rs4663402 were significantly associated with the risk of HCC, in which the ATG10 10514231 was the strongest factor. Bioinformatics analysis of TCGA database showed that mRNA expression of the ATG5, ATG10, ATG12, and ATG16L1 genes in HCC tissues was higher than that in normal tissues [111]. 

**Figure 1:**
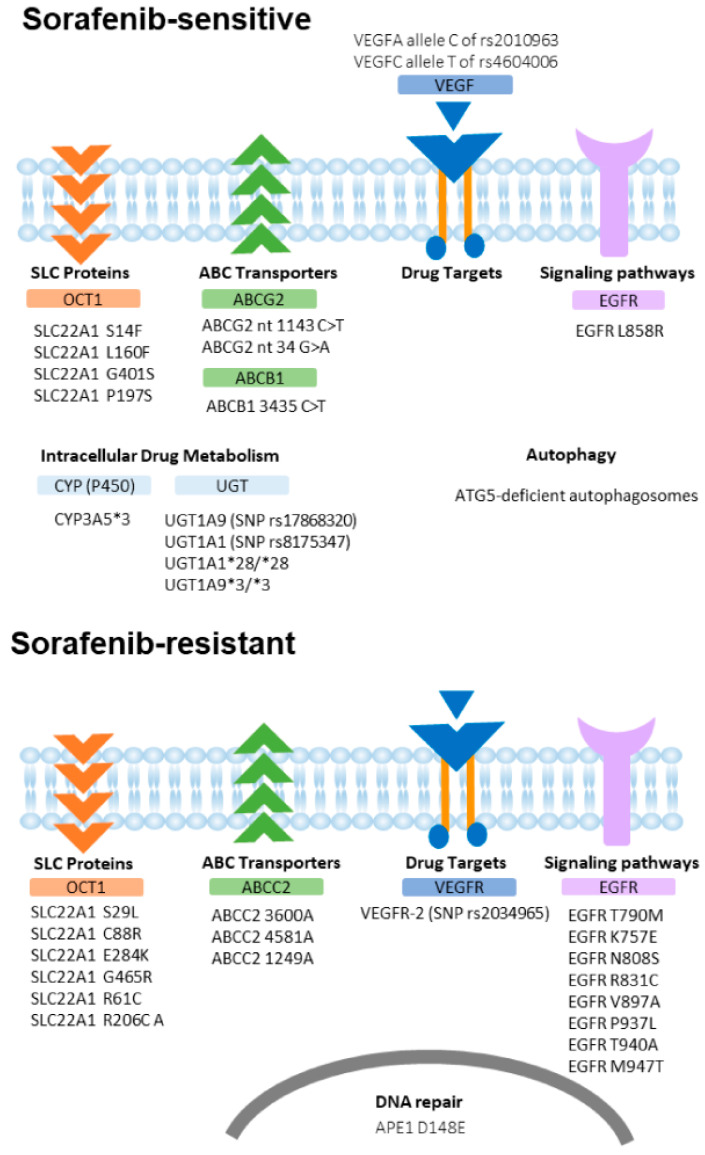
Genetic polymorphisms and variants on drug-resistant molecules in hepatocellular carcinoma. Several reported DNA polymorphisms and protein variants reportedly played role in sorafenib sensitivity (upper panel) and sorafenib resistance (lower panel), including in ABC transporters, SLC proteins, autophagy, and DNA repair molecules.

Listed in Figure 1 and Table 1 are the specific genetic variants and mechanisms affecting sorafenib chemoresistance. 

### 2.8. Hepatic Cancer Stem Cells

Subpopulations of cancer cells coexist in primary tumors like HCC, with each clonal variant having different sensitivity to chemotherapy [112]. In the cellular context, intratumorally heterogeneity is closely associated with the cancer stem cells (CSC) populations. The CSC is one of the main players in initiating cancer still maintaining normal stem cell properties such as self-renewal and multi-differentiation into multiple cell types. To identify and isolate HCC CSC populations, at least 12 different phenotypical CSC markers (e.g., CD133/Prominin-1, CD90/Thy-1, EpCAM/CD326, CD13, and ABCG2) have been used. The combination of these markers determines additional subpopulations in one CSC population, resulting in a wide variety of hepatic CSC phenotypes, also within a single tumor [113]. The CSC are characterized by high resistance to chemotherapies, due to high expression of the ABCG2/BCRP transporter, the molecular determinant of the so-called side population [114,115,116]. 

Recent evidence showed the relevance of the hepatic CSC in sorafenib resistance, mostly in cellular models. Prolonged treatment with sorafenib led to the enrichment of hepatic CSCs, while the general population of differentiated cancer cells undergoes growth suppression [117,118,119,120,121,122]. It should be noted that combination therapy between sorafenib and inhibitors against the targeted signaling pathway (P13K/Akt/mTOR) could overcome drug resistance. In isolated CSCs and xenograft models, cotreatment with sorafenib and MK2206, a PI3K/Akt signaling inhibitor was reported to reduced pAkt and pERK expression [123]. The sequential treatment of rapamycin followed by sorafenib also reduced the presence of the CSC as well as their sphere formation capacity [118]. 

The importance of intratumoral heterogeneity in hepatic CSC in clinical specimens had been demonstrated. Colombo et al. isolated differentially expressed CSC markers identified from three phenotypically distinct cell populations coming from a single HCC lesion. The heterogeneous cell populations showed a difference in doubling times, drug resistance, and tumorigenic potential [124]. Another work of Gao et al. clearly showed the effect of cellular intra-heterogeneity to sorafenib resistance. In this study, 55 HCC regions from 10 patients were subjected to histologic analysis, isolation, and multiplication of cancer cells, obtaining hundreds of so-called patient-derived primary cancer cells (PDPC). Following PDPCs insensitivity to sorafenib and the absence of genomic markers for sorafenib sensitivity, cells were profiled based on the expression of druggable genes and druggable genomic alterations in intratumor subregions. The use of the biomarker-oriented heterogeneity to assess drug sensitivity will not only drive to effective treatment but might be pivotal in overcoming resistance to therapy and cancer progression [125]. Despite this challenge of heterogeneity in CSC, several CSC markers are found associated with drug response. The overexpression of the CSC markers CD133 and CD90 in HCC specimens was associated with a poorer response to sorafenib [126,127]. In a CD133+ xenograft tumor, inhibition of JNK reduced tumor growth, suggesting that JNK activity may be considered as a new predictive biomarker for response to sorafenib treatment [126]. 

## 3. Future Perspectives

Following the Darwinian theory of expansion in cancer, drug-resistant cells will have a survival advantage and establish a tumor with a genomic profile more resistant to the initial treatment [128]. Therefore, the study of MOCs is important to develop new drugs, to discover biomarkers for treatment, and to determine combination drug regimens to enhance drug sensitivity. While the ability of sorafenib to be multi-targeted gives it an upper hand in providing an effect, the same multi-targets are bound for polymorphism promoting heterogeneity leading to chemoresistance and treatment failure. These genetic variants are also evident in drug transportation systems, signaling pathways, drug metabolism and cellular processes which are important in understanding resistance and sensitivity to the drug. 

**Figure 2:**
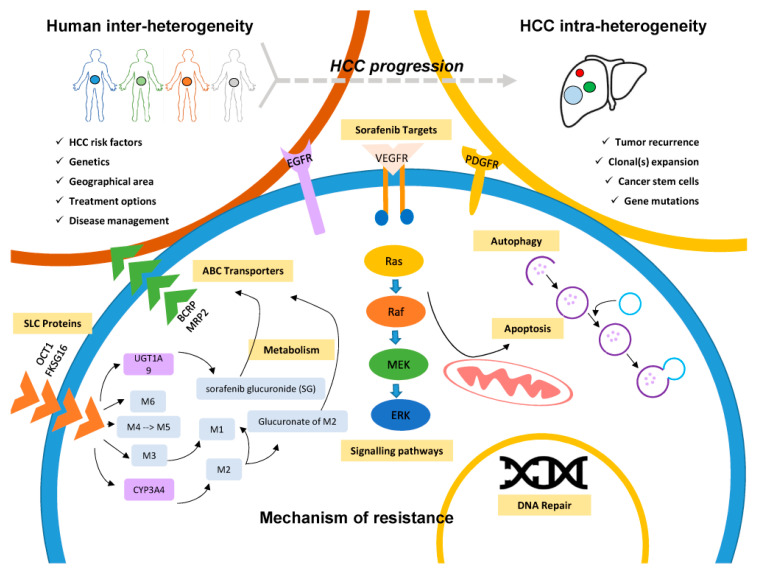
Mechanisms of sorafenib resistance. Sorafenib as an anticancer drug is transported into the cell through SLC proteins (e.g., OCT1). Inside the cell, the drug is broken-down via phase 1 cytochrome P450 3A4 (CYP3A4) and phase 2 UDP glucuronosyltransferase 1A9 (UGT1A9) mediated metabolism to form the M1-M8 metabolites [84]. Sorafenib glucuronide (SG) is excreted out of the cell by a process mediated by ABC transporters. Aberrations in the expression of SLC proteins and ABC transporters, as well as genetic polymorphisms in metabolizing enzymes can control intracellular concentration, subsequently influencing drug efficacy. Sorafenib is a drug designed to target multiple tyrosine kinase inhibitors such as VEGF, PDGF, and EGF that activate downstream signaling pathways such as the Ras/Raf/MEK/ERK pathway. Overexpressed drug targets and perpetually activated signaling pathways will promote cell proliferation and survival, therefore promoting chemoresistance. Cellular processes such as DNA repair, apoptosis, and autophagy can redirect their effect to promote cell proliferation and survival rather than cell death.

The challenge of intratumor heterogeneity adds to the difficulty of observing a uniform drug response across a single tumor. Expanding information both on specific target studies or on high-throughput analysis in human HCC specimens have consistently demonstrated that various human genetic variants and dysregulated molecules affected the MOCs. The genetic variants found in HCC were associated with variation in transport mediated drug intake and efflux, metabolism catalytic enzymes, signaling pathways, changes in the DNA repair machinery, and autophagy (Figure 2). These variants could serve as potential markers to identify patients that might benefit from a sorafenib treatment, a combination of drug regimens, or a switch to another type of therapies (e.g., immunotherapy). 

The various genetic heterogeneity factors affecting chemoresistance in HCC treatment, particularly in sorafenib, are presented in this review. Since sorafenib shares its molecular targets with other multikinase inhibitors (e.g., regorafenib and lenvatinib which targets also VEGFR1-3 and PDGRFβ), this information can also be useful in assessing MOCs in other molecularly targeted therapies.

The development in genetics, molecular, and cellular biology, allows taking advantage of various biological models of molecular targeting. Cloning, site-directed mutagenesis, and CRISPR/Cas9 technologies can be used to target a specific molecule in available immortalized cancer cell lines, or even patient-derived cells. The latter may provide a specific and individualized therapy based on tumor characteristics of an individual. The use of 3-dimensional cultures, spheroids, and organoids would allow investigation of drug response considering other organ components and characteristics (3D- architecture, cell-to-cell, cell-to-matrix interactions, cellular heterogeneity, and tumor microenvironment), as reviewed in [129]. It is also important not to forget the use of in vivo models, especially rodents, such as xenografts, transgenic models, and chemically-induced animals, that can mimic the natural history of HCC in humans. These tools can advance our understanding of drug-resistant protein markers, cellular and organ chemosensitivity to drugs, which can be translated into clinical practice.

Since advanced HCC is commonly accompanied by a high degree of heterogeneity, HCC management should focus on early diagnosis and a rapid treatment. It would remain the best strategy to avoid vast cancer heterogeneity and cancer cell resistance, thus leading to a more successful therapy [130]. It is necessary to consider multitarget approaches because of the very diverse nature of the tumor and the involvement of multiple cellular processes. Computational approaches in combination with -omics (e.g., transcriptomics, metabolomics) and various disease models cited above may give us more tools to manage sorafenib resistance in HCC. The future will keep geneticists, molecular biologist, pharmacologists and clinicians busy.

## 4. Conclusions

Sorafenib remains one of the limited treatment options for advanced HCC patients. Herein we review the involvement of various key players in sorafenib transport, metabolism, and other resistance factors, with particular emphasis on the HCC genetic heterogeneity. The genetic variants and mutants described in this paper will have potential role in determining drug response, not only in sorafenib therapy, but also in other molecular targeted therapies, leading to improvement in HCC treatment and management.

## Figures and Tables

**Table 1:** Mechanisms of resistance against sorafenib in HCC.

Factors	Mechanism Contributing to Resistance	Molecular Markers	Effect to Chemotherapy or Cellular Function
**Drug Concentration**			
Drug efflux via ABC transporters	Overexpression of specific ABC transporters; genetic polymorphism	ABCB1; ABCC1; ABCC2; ABCC3; ABCG2	Decreased intracellular drug concentration
Drug uptake via SLC transport proteins	Downregulation of SLC transport proteins; genetic polymorphism; localization (plasma membrane expression only)	SLC22A1; SLC22A3; SLC46A3	Decreased intracellular drug concentration
**Cellular Processes**			
Alteration of target molecule	Overexpression of drug target molecules; genetic polymorphism and mutation	Raf; VEGF	Promote angiogenesis; sustained activation of signaling pathway promoting cellular proliferation and growth
Alteration in signaling pathway	Overexpression and sustained activation of signaling pathway molecules; genetic polymorphism and mutation	EGFR Ras/Raf/MEK/ERK pathway)	Sustained activation of signaling pathway promoting cellular proliferation and growth
Intracellular drug metabolism	Genetic polymorphism in metabolizing enzymes	CYP3A5; CYP450; UGT1A9; UGT1A1	Decrease in drug metabolism
DNA repair	Overexpression sustained activation of DNA repair proteins; genetic polymorphism	APE1	Promote DNA repair therefore inhibiting apoptosis
Autophagy	Stress response that function as double-edged; Agonist or antagonist to treatment	ATGs	Promote cell survival and proliferation or otherwise
**Intra- and Inter-Cellular Properties**			
Cancer Stem Cells	Presence of cancer stem cell properties poses survival advantage	CD133, CD90, EpCAM, ABCG2	Failed therapy
Tumor genetic heterogeneity	Tumoral heterogeneity dictates differential sensitivity of cells to chemotherapy within a tissue; cells ability for clonal expansion allows survival of resistant cells	-	Failed therapy
